# Repeatable tract-based diffusion metrics on a portable 64 mT MRI: a foundation for global population neuroscience

**DOI:** 10.3389/fnimg.2026.1845201

**Published:** 2026-07-30

**Authors:** William Royer, James Gholam, Mara Cercignani, Derek K. Jones, Emre Kopanoglu

**Affiliations:** 1 Cardiff University Brain Research Imaging Centre (CUBRIC), Cardiff University, Cardiff, United Kingdom; 2 School of Psychology, College of Biomedical and Life Sciences, Cardiff University, Cardiff, United Kingdom

**Keywords:** democratizing MRI, diffusion tensor imaging, low-field MRI, repeatability, scalable MRI, tractography

## Abstract

Diffusion MRI underpins much of modern population neuroscience, yet scanner access remains concentrated in high-income settings, excluding the genetic, developmental, and disease diversity needed for generalizable discovery. Portable low-field MRI systems (field strengths below 0.1 T) offer a cheaper, infrastructure-light alternative that could extend diffusion imaging to under-represented populations, but only if the measurements are sufficiently repeatable. Here we provide the first systematic test-retest evaluation of tract-based diffusion tensor MRI (DT-MRI) metrics at 64 mT. Ten healthy participants were scanned at two time-points (median interval 13 days) with an 18-direction (*b* = 900s/mm^2^) diffusion protocol and a *T*_2_-weighted structural sequence on a portable 64 mT scanner. After correction for distortions and gradient imperfections, diffusion tensors were estimated and constrained spherical deconvolution was performed to enable bundle-specific tractography. Tract averaged fractional anisotropy, mean diffusivity, and radial diffusivity were extracted from a selection of white-matter tracts spanning projection, association, and commissural fiber categories. Bland-Altman analysis and within-subject coefficients of variation indicated strong scan-rescan agreement, while intraclass correlation coefficients were variable across tracts and metrics. Compared with high-field data, coefficients of variation were 3–10 times larger and standard deviations in the means were approximately an order of magnitude higher. Despite this, statistical power calculations yielded feasible sample-size estimates for detecting group differences in a two-tailed *t*-test: for a 4% difference in means, approximately 25 participants per group were sufficient for the majority of tracts in MD and RD, and around 65 for FA. Although absolute DT-MRI metric values diverge from high-field references due to partial volume effects and noise-floor bias, continued advances in acquisition, reconstruction, and processing are expected to further narrow this gap. The results demonstrate that portable 64 mT MRI can deliver repeatable DT-MRI metrics, establishing a foundation for democratizing advanced neuroimaging and enabling population-scale studies in regions that have historically been excluded from brain research.

## 1 Introduction

Diffusion MRI offers a non-invasive window into brain microstructure and connectivity, supporting neuroepidemiological research. However, the vast majority of brain imaging data currently lack diversity (Wogu et al., 2025) and do not sufficiently represent populations in low- and middle-income countries (LMICs), owing to an economically driven gap in access to MRI. For instance, in 2018, West Africa had only 84 functional MRI units for 372 million people—roughly 1 scanner per 5 million inhabitants across the 16 countries surveyed (Ogbole et al., 2018). Within this region, Ghana and Nigeria accounted for 72 of the 84 units (86%), while Benin Republic, Mali, Liberia, Niger, and Sierra Leone had none, despite a combined population of 62.2 million. In contrast, the USA and Japan had approximately 196 and 276 MRI units per 5 million inhabitants, respectively (OECD, 2019). Moreover, many units in LMICs become nonfunctional once initial service contracts provided with the scanner at purchase lapse, creating a growing “medical graveyard” of unusable systems (Jones et al., 2025).

This inequity in access to advanced neuroimaging has resulted in the under-representation in neuroscience of populations in LMICs that hold unique value for advancing discoveries in neuroscience. For example, Africa's unmatched human genetic diversity has already demonstrated that findings are not always generalizable across populations (Hendrie et al., 2014; Neu et al., 2017), and the continent's diverse ecosystems offer rich opportunities for comparative research. Furthermore, African populations experience higher incidences of brain injuries and infections than those in the Global North, alongside developmental challenges such as malnutrition (Donald et al., 2022). Greater inclusions of populations from the Global South is therefore essential for building a more complete model of brain health and for advancing the fields of neurogenetics and infectious disease neurology (Jones et al., 2025).

Low-field permanent magnet MRI systems [corresponding to field strengths of 0.01 T to 0.1 T according to recent classification guidelines from the International Society for Magnetic Resonance in Medicine safety committee (Kopanoglu et al., 2025)] are emerging as a more accessible alternative that could help expand the neuroimaging community in LMIC settings, and leverage the unique diversity to advance the field. Because they use permanent magnets instead of superconducting ones, low-field systems are cheaper to buy, easier to install and require no cryogenics. Their small footprint and low power demands enable true portability.

At low field strengths, low signal-to-noise ratios (SNR) typically result in longer acquisitions, and yield images with lower resolution and contrast. These limitations have constrained both clinical and research applications at low field, particularly for the SNR-demanding quantitative diffusion metric maps and tractography. However, advances in image reconstruction, acquisition strategies, and post-processing have greatly expanded what is achievable with these instruments. Notably, recent work (Gholam et al., 2026b) has demonstrated reasonable agreement between 3 T and 64 mT tractography.

Building on these advances, the present work evaluates the scan-rescan repeatability of diffusion MRI metrics at 64 mT. Establishing reliable diffusion measurements at low field is a prerequisite for population-scale neuroscience and for democratizing advanced neuroimaging in regions that have historically been excluded. Here we provide the first systematic test-retest evaluation of diffusion tensor metrics at 64 mT and show that, despite reduced SNR and FA values, repeated measurements are sufficiently stable to support group-level inference.

## 2 Methods

### 2.1 Acquisition

Ten healthy participants (5M/5F) with ages between 18 and 32 (mean = 27.4 ± 4.45 SD) were scanned with the Hyperfine Swoop^®^ (software version RC9.0.0_BETA1) system at two time-points (median interval: 13 days). The 70-min protocol included:

1. A single *b* = 0s/mm^2^ volume.

2. A 2 mm-isotropic *T*_2_-weighted structural image.

3. 18 diffusion-weighted volumes, each along a different direction, at *b* = 900s/mm^2^. These were acquired with a multi-shot fast-spin-echo sequence at a 3 mm-isotropic resolution, with TE = 84 ms and TR = 800 ms. Each volume used 212 shots containing 70 echoes each.

4. Short calibration scans to reset the scanner *f*_0_ (center frequency), placed after the *b* = 0s/mm^2^ and *T*_2_ acquisitions, then between each diffusion volume. This was done to account for frequency shifts due to *B*_0_ (main magnetic field) drifting as a result of the scanner temperature increasing.

All acquisitions used a 3D multi-shot fast-spin-echo read out, see related work (Gholam et al., 2026b) for further sequence details.

### 2.2 Processing

Images were reconstructed using the vendor's proprietary deep learning algorithm. All diffusion volumes and the *T*_2_-weighted structural image were registered (nonlinear registration) to the *b* = 0s/mm^2^ volume using ANTs (Tustison et al., 2021). The affine transformations from the registration were also used for rotating the b-vectors to re-align them with the image, preserving correct orientational information (Leemans and Jones, 2009). A bias field correction was then applied to address a previously reported directional bias in the diffusion signal caused by encoding gradient field imperfections. This was done by applying a Fermi spectral filter to form smooth individual bias images, then computing an average bias image, and normalizing the individual bias fields to return an unbiased image (O'Halloran et al., 2013; Gholam et al., 2026a). A diffusion tensor (Bihan et al., 2001) was estimated in each brain voxel with *dtifit* from FSL (Jenkinson et al., 2012), using a linear least-squares fit. The brain mask - which excluded the skull and cerebrospinal fluid - was computed using Freesurfer's *synthstrip* tool (Hoopes et al., 2022; Kelley et al., 2024).

The *T*_2_-weighted image was super-resolved to a synthesized 1 mm-isotropic *T*_1_-weighted image using *synthSR* (Iglesias et al., 2021, 2023). To extract a white-matter mask, the resulting image was segmented using Freesurfer's *samseg* tool (Puonti et al., 2016), which gives up to five tissue types: cortical gray matter, subcortical gray matter, white matter, cerebrospinal fluid, and pathology. This mask was used to constrain the estimation of the single-fiber response function using a recursive calibration approach (Tax et al., 2014), identifying voxels likely to contain a single-fiber population and characterizing their typical diffusion signal. With MRtrix3, this response function was then used for single-tissue constrained spherical deconvolution to obtain fiber orientation distribution functions (fODFs) (Tournier et al., 2019). The peaks of the fODFs were extracted and served as input to TractSeg (Wasserthal et al., 2018), a bundle-specific tractography method that segments anatomically defined white-matter tracts directly from orientation information without requiring whole-brain tractography as an intermediate step. Tracts were visually overlaid on the super-resolved *T*_1_ with the scilpy toolbox (Renauld et al., 2026), and volume renders were made using VIBRANT (Kraaijeveld et al., 2025), a cinematic rendering tool. Bundles presented in figures are colored by principal eigenvector orientation (Pajevic and Pierpaoli, 1999).

### 2.3 Population level maps

To demonstrate the feasibility of population-level analyses, group-average maps were produced from the full dataset. A template FA image was first constructed using *antsMultivariateTemplateConstruction2* (Tustison et al., 2021). The transformations mapping each input image to the template space were then applied to the individual diffusion tensor maps using ANTs (*antsApplyTransforms* and *ReorientTensorImage*), and the registered tensor maps were averaged to produce an average principal eigenvector map.

### 2.4 Repeatability

#### 2.4.1 Tract selection

Repeatability of FA, MD, and RD values was investigated within a selection of white-matter tracts including projection, commissural, and association fibers : the arcuate fasciculus (AF), anterior thalamic radiation (ATR), corpus callosum posterior midbody and splenium (CC 5 and CC 7 respectively), corticospinal tract (CST), cingulum (CG), and inferior fronto-occipital fasciculus (IFO). These tracts are of particular relevance in neuroscience, covering motor function (CST), language (AF), thalamo-cortical connectivity (ATR), interhemispheric communication (CC), and limbic and cognitive function (CG).

#### 2.4.2 Statistical assessment

Tract-averaged FA, MD, and RD values were extracted from each tract's region-of-interest mask outputted by TractSeg. Test-retest agreement was assessed with a Bland-Altman analysis (Altman and Bland, 1983). Within-subject variance was quantified with the within-subject coefficient of variation (wsCV). Intra-participant versus inter-participant variance was quantified with the intraclass correlation coefficient (ICC(2,1)), computed with the Pingouin package (Vallat, 2018) in Python using a two-way random-effects model with absolute agreement (Koo and Li, 2016; Koller et al., 2021; Melzer et al., 2020). Using this same package, 95% confidence intervals and a multiple comparisons false discovery rate *p* value correction using the Benjamini/Hochberg method were also calculated (McGraw and Wong, 1996; Benjamini and Hochberg, 1995).

### 2.5 Power analysis

The statistical power of detecting group differences was assessed by estimating the sample sizes required to achieve a significant result in an independent-samples two-tailed *t*-test, with significance level α = 0.05 and statistical power 1−β = 0.8. The effect size was defined by Cohen's *d*:


$$\begin{array}{l} d=\frac{{\bar{x}}_{1}-{\bar{x}}_{2}}{\sigma } \end{array}$$ (1)

where ${\bar{x}}_{1}$ and ${\bar{x}}_{2}$ are the group means and σ is the total standard deviation, combining inter- and intra-participant variance components. Mean difference of 2 and 4% were chosen, consistent with other MRI repeatability studies (Melzer et al., 2020; Koller et al., 2021). Equal variance was assumed for both groups. The sample size calculations were performed with a statistical power analysis package (Huijzer, 2021) in Bezanson et al. (2017).

## 3 Results

### 3.1 Individual data points

**Figure 1:**
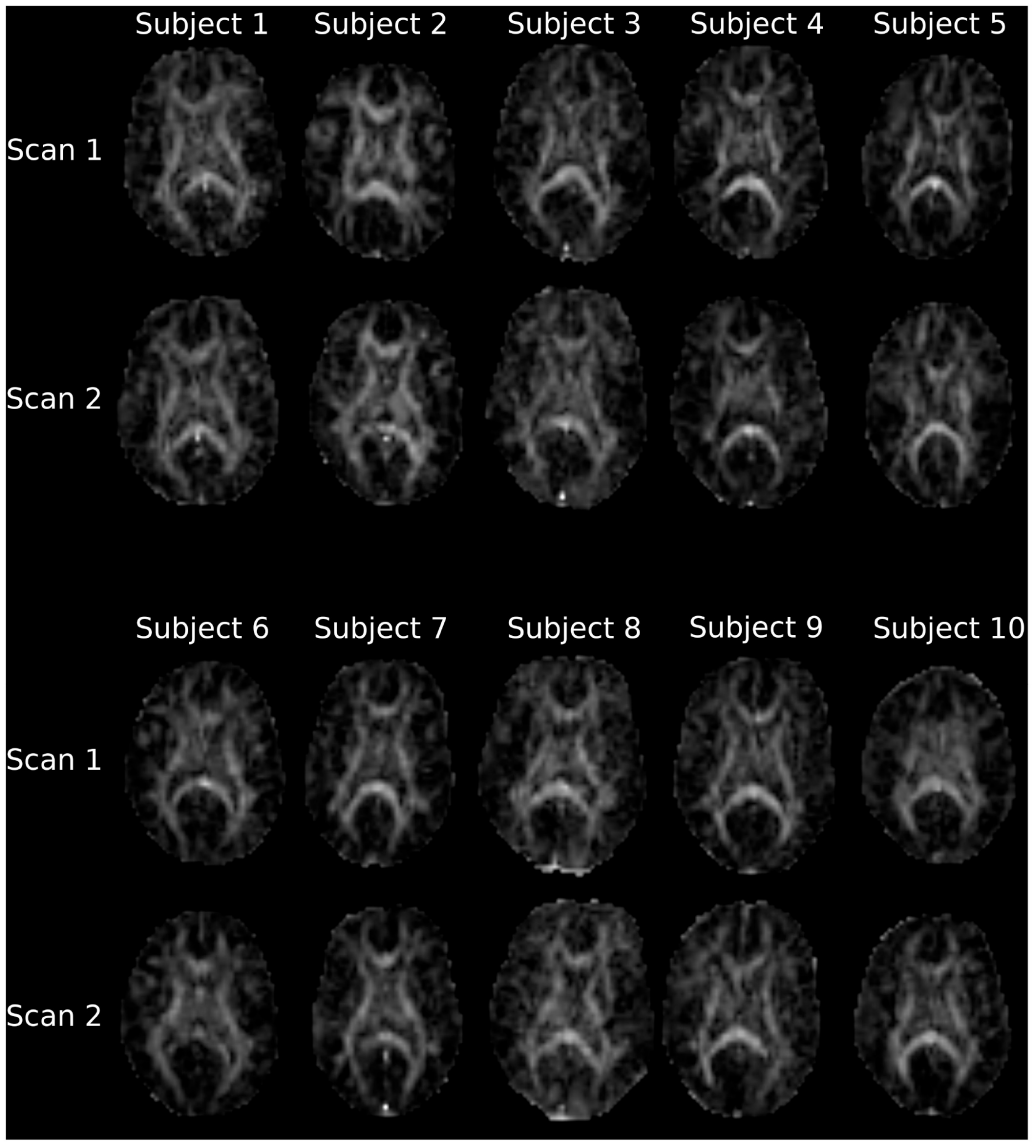
FA images for all 10 participants.

**Figure 2:**
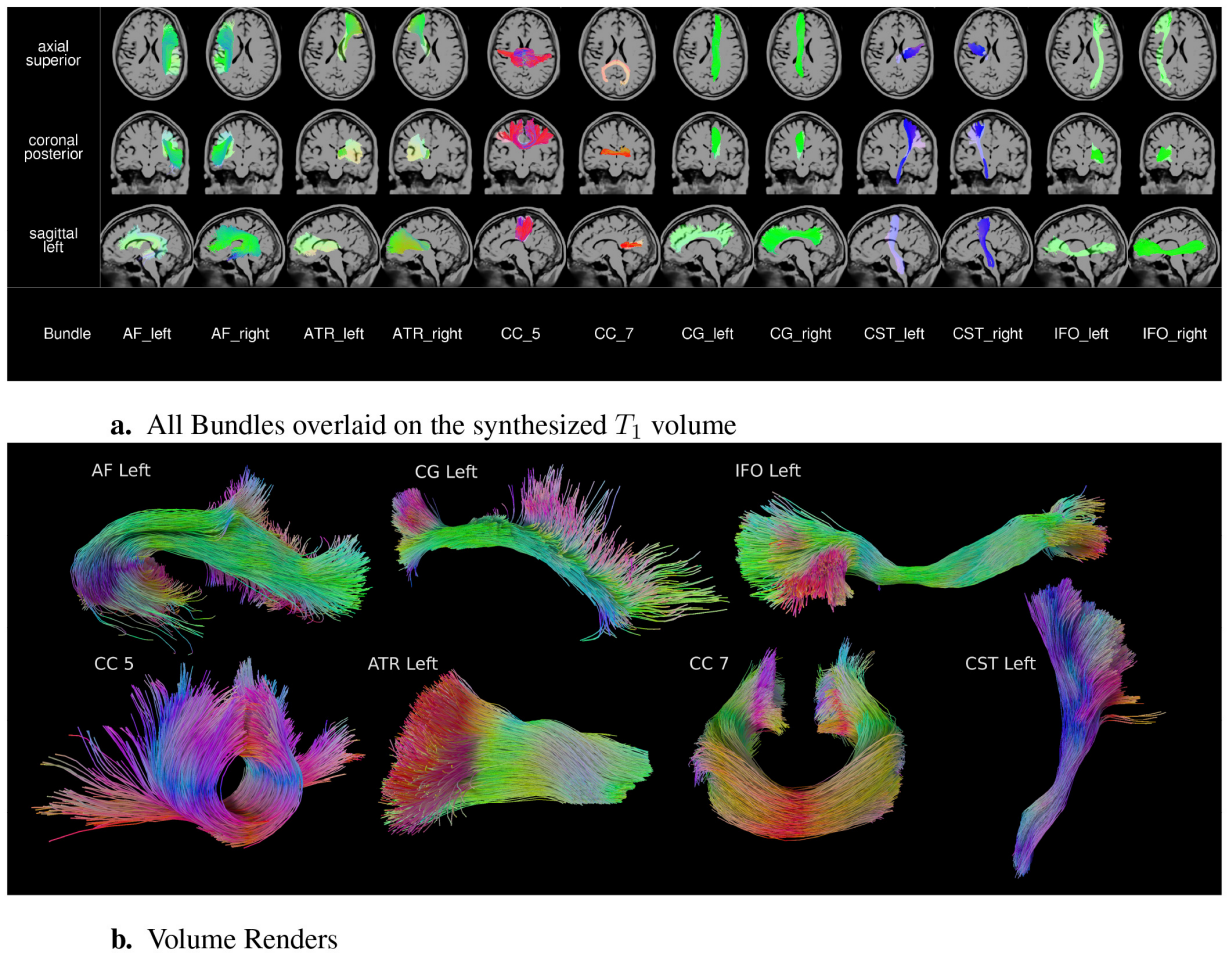
Bundle Segmented Tractography results, colored by principal eigenvector orientation. **(a)** All Bundles of interest for a participant overlaid on their synthesized *T*_1_ volume. **(b)** Volume renders for the selection of tracts in a participant, only including the left hemisphere for bilateral tracts.

Figure 1 illustrates the scan-rescan FA quality. Large white-matter bundles are robustly resolved, whereas smaller peripheral tracts remain inconsistent. Similarly tractograms shown in Figures 2a and b visually demonstrate the standard of bundle segmented tractography at low-field using improved processing methods.

### 3.2 Population level maps

**Figure 3:**
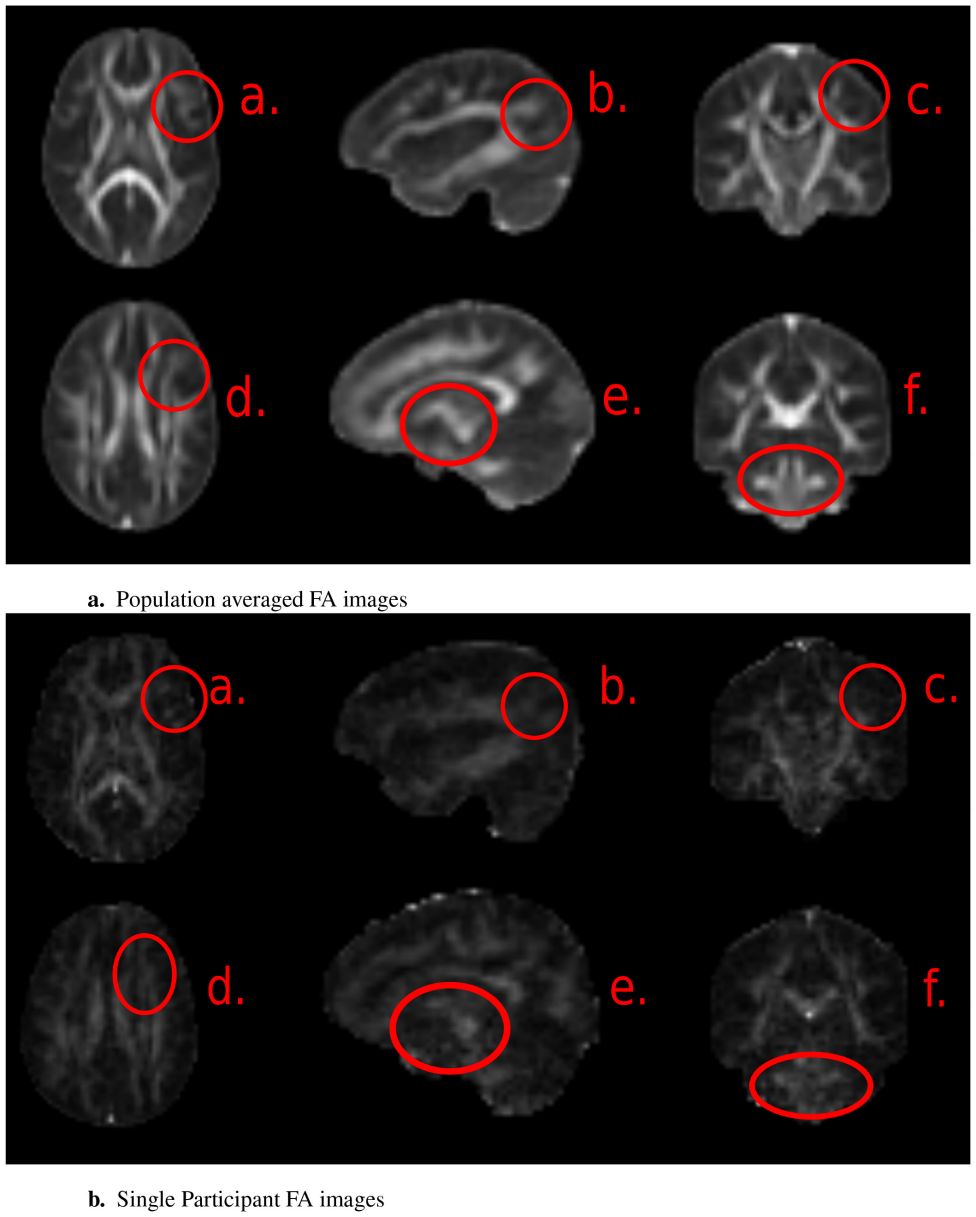
Comparison across multiple slices and orientations of the population averaged FA map with a single participant FA map. Features that are recovered when averaging over a population U fibers (a.), Superior portion of the arcuate fasciculus (b.), corona radiata and internal capsule (c.), internal capsule (d.), midline ventral diencephalon (e.), spinal cord and cerebellar peduncles (f.). **(a)** Population averaged FA images. **(b)** Single Participant FA images.

**Figure 4:**
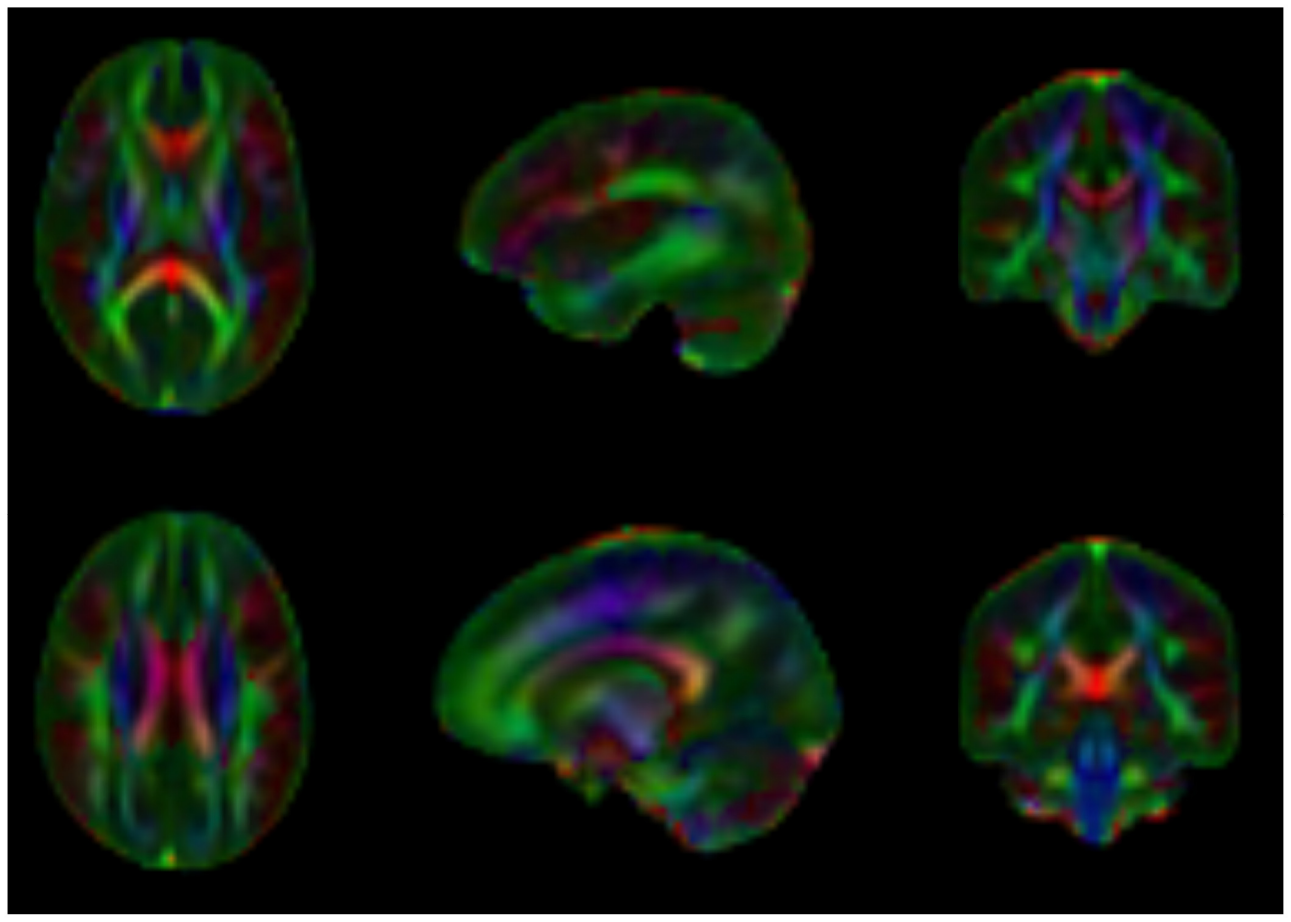
Population averaged principal eigenvector colored FA maps in axial,sagittal, and coronal orientations.

Substantially lower noise maps are obtained when averaging across the population, as shown for FA in Figure 3. Previously unresolved features become apparent, particularly in the periphery of the brain. These newly resolved features include U fibers, superior portion of the arcuate fasciculus, corona radiata and internal capsule, internal capsule, midline ventral diencephalon, spinal cord and cerebellar peduncles. The population averaged maps could enable visual comparisons of small regions of interest that may differ between populations. The averaged color FA in Figure 4 shows slightly worse feature separation than the averaged FA map, reflecting the added difficulty of averaging orientational information across subjects.

### 3.3 Repeatability

The mean, standard deviation, and wsCV averaged over all participants are shown for FA, MD, and RD in each tract in Table 1.

**Table 1:** Scan-rescan repeatability of diffusion tensor metrics across white-matter tracts.

Metric	Tract	Mean	SD	wsCV(%)	ICC	ICC 95% CI	ICC *p*	ICC *q*
FA	CST left	0.19	0.014	3.6	0.62	[0.02, 0.89]	0.025	0.108
MD		1.05	0.04	2.6	0.32	[-0.41, 0.78]	0.182	0.188
RD		0.95	0.04	3.0	0.33	[-0.4, 0.78]	0.176	0.186
FA	CST right	0.19	0.012	3.4	0.59	[-0.01, 0.88]	0.031	0.111
MD		1.07	0.04	2.3	0.44	[-0.28, 0.83]	0.101	0.137
RD		0.96	0.04	2.4	0.47	[-0.23, 0.84]	0.085	0.122
FA	AF right	0.13	0.01	3.0	0.76^*^	[0.29, 0.94]	0.004	0.037
MD		1.14	0.05	2.2	0.51	[-0.19, 0.85]	0.066	0.115
RD		1.06	0.05	2.3	0.53	[-0.15, 0.86]	0.055	0.115
FA	AF left	0.13	0.01	2.8	0.82^*^	[0.42, 0.95]	0.001	0.016
MD		1.08	0.05	2.8	0.51	[-0.18, 0.86]	0.064	0.115
RD		1.01	0.05	3.0	0.54	[-0.15, 0.86]	0.053	0.115
FA	ATR right	0.15	0.01	4.1	0.58	[-0.03, 0.88]	0.034	0.111
MD		1.24	0.05	2.4	0.39	[-0.35, 0.81]	0.135	0.155
RD		1.14	0.05	2.6	0.42	[-0.31, 0.82]	0.114	0.145
FA	ATR left	0.15	0.011	3.7	0.7	[0.16, 0.92]	0.01	0.075
MD		1.2	0.05	2.6	0.51	[-0.19, 0.85]	0.066	0.115
RD		1.11	0.06	2.8	0.52	[-0.17, 0.86]	0.06	0.115
FA	IFO left	0.16	0.012	4.1	0.65	[0.08, 0.9]	0.018	0.106
MD		1.17	0.05	2.7	0.38	[-0.37, 0.8]	0.144	0.158
RD		1.08	0.05	3.1	0.39	[-0.35, 0.81]	0.137	0.155
FA	IFO right	0.17	0.011	3.4	0.64	[0.03, 0.9]	0.021	0.108
MD		1.2	0.05	2.3	0.44	[-0.28, 0.83]	0.103	0.137
RD		1.09	0.05	2.4	0.41	[-0.31, 0.82]	0.117	0.145
FA	CG right	0.13	0.009	4.7	0.5	[-0.2, 0.85]	0.071	0.115
MD		1.2	0.04	2.0	0.53	[-0.17, 0.86]	0.059	0.115
RD		1.12	0.04	2.3	0.52	[-0.18, 0.86]	0.063	0.115
FA	CG left	0.14	0.011	3.9	0.62	[-0.01, 0.89]	0.027	0.108
MD		1.17	0.04	2.0	0.49	[-0.21, 0.85]	0.074	0.115
RD		1.08	0.04	2.3	0.49	[-0.22, 0.85]	0.077	0.116
FA	CC 5	0.17	0.016	2.5	0.9^*^	[0.65, 0.97]	<0.001	0.004
MD		1.07	0.04	2.2	0.39	[-0.33, 0.81]	0.133	0.155
RD		0.98	0.04	2.4	0.49	[-0.2, 0.85]	0.073	0.115
FA	CC 7	0.22	0.03	4.4	0.84^*^	[0.5, 0.96]	<0.001	0.007
MD		1.16	0.04	2.8	0.31	[-0.42, 0.77]	0.195	0.195
RD		1.03	0.05	3.0	0.48	[-0.18, 0.84]	0.073	0.115

Mean and SD are computed from session-averaged values per subject. MD and RD are reported in units of × 10^−3^ mm^2^/s. wsCV: within-subject coefficient of variation averaged over all participants; ICC: intraclass correlation coefficient (ICC (2,1) two-way random, absolute agreement). ICC *q* shows the corrected *p* value for multiple comparisons using Benjamini/Hochberg false discovery rate correction. Significant ICC values that survive the correction are marked with an asterisk.

**Figure 5:**
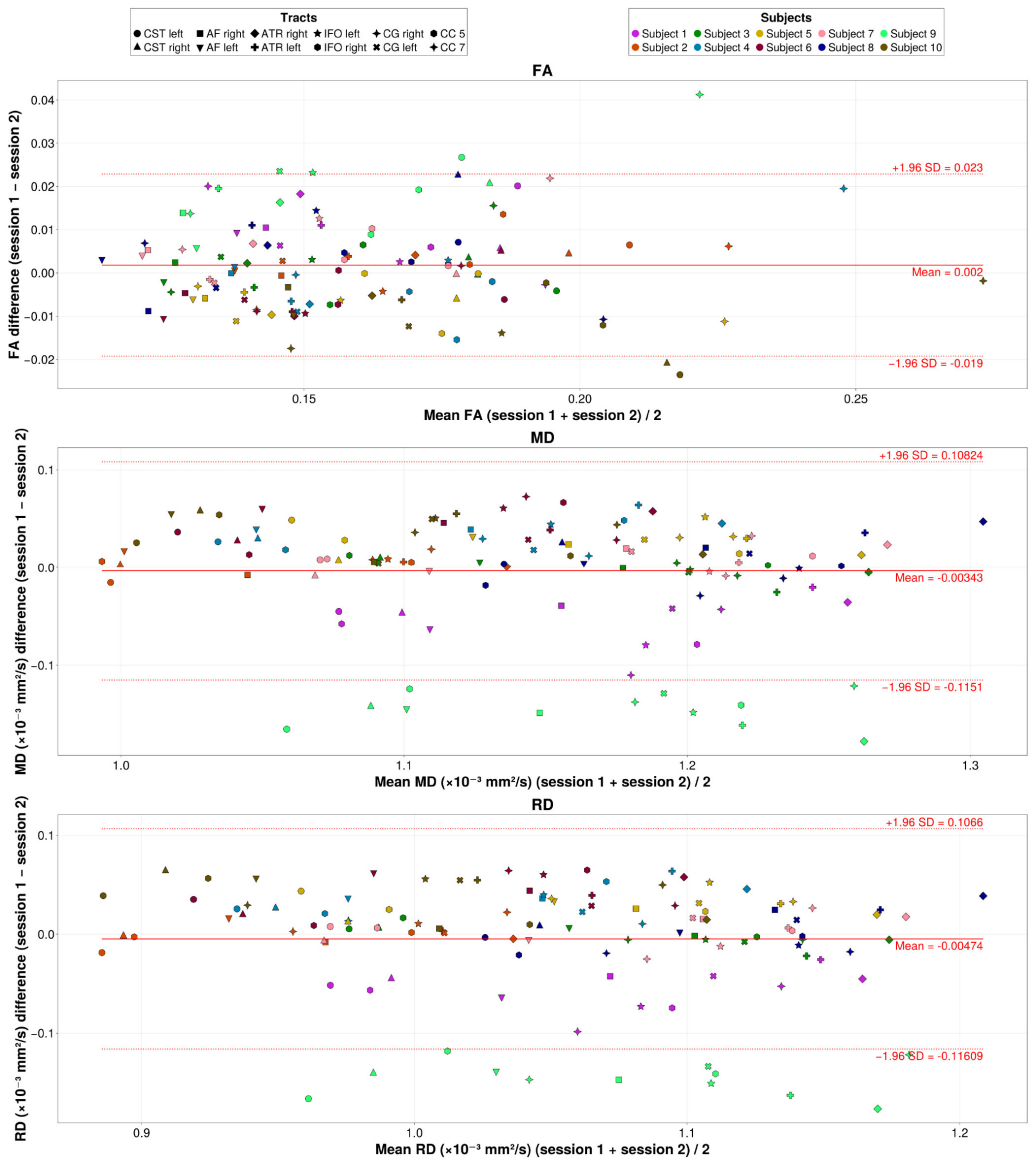
Bland-Altman analysis plots for FA, MD, and RD, with marker shape and color representing different tracts and participants respectively.

A Bland-Altman analysis using the differences of these means is shown in Figure 5. Virtually all data-points lie within the limits of agreement, suggesting strong repeatability of these DT-MRI metrics in this selection of tracts. The points that do not lie within this interval for MD and RD all come from the same participant, where one of the scans had abnormally high diffusivity measures.

**Figure 6:**
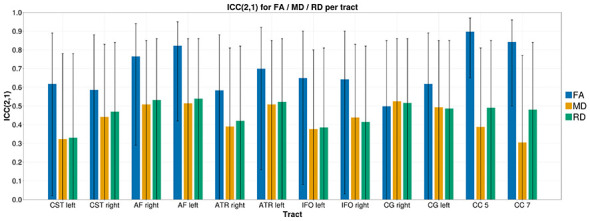
Intraclass correlation coefficient (ICC) for FA, MD, and RD for the selection of white matter tracts. 95% confidence interval bars shown, and to improve readability of the figure, the vertical axis lower limit was set to 0.

The ICC scores in Figure 6 and Table 1 reveal a varying reliability between repeat scans depending on which tract and metric is investigated. For MD and RD, all tracts show poor (below 0.5) to moderate (0.5 to 0.75) ICC. In contrast, good (0.75 to 0.9) ICC reliability is obtained for FA in both AF left and right, as well as in both CC regions. All other tracts have moderate scores for FA.

The generally non-significant ICC *p* values indicate that inter-subject variability was often not sufficiently larger than measurement variability to be detected reliably in this cohort. The 95% confidence intervals reported in Table 1 and shown in Figure 6 indicate considerable uncertainty in the ICC estimates. The multiple comparisons corrections removed statistical significance from 7 out of 11 ICC *p* values, leaving FA in the corpus callosum (both segments) and arcuate fasciculus (bilaterally) as the only reliable combination of tract and metric.

### 3.4 Sample size calculations

**Figure 7:**
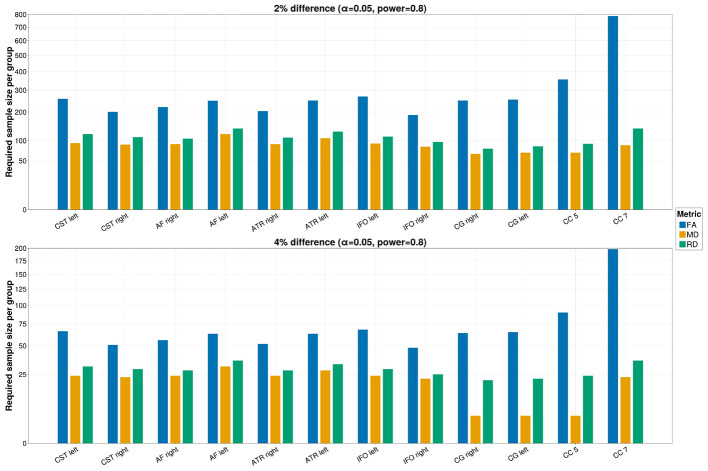
Per group sample size estimates for significantly detecting 2% and 4% differences in the mean in a two tailed *t*-test.

Figure 7 shows the estimated sample sizes per group required in a 2 tailed independent groups *t*-test, with differences in the mean of 2% and 4%. Sample sizes required for detecting significant differences in MD and RD are substantially smaller than those required for FA, with a high variability in estimates between different bundles.

## 4 Discussion

### 4.1 Deviation from high field values

The FA values reported in Table 1 are consistently lower than values usually obtained from high field measurements. For instance, the splenium segment of the CC, the tract showing the highest FA in this work, has a FA value of 0.22, as opposed to 0.81 in a 3 T measurement in healthy adults (Mehta and Sharma, 2023). This can be explained by the acquisition parameters. The long echo train increases *T*_2_ decay, broadening the point spread function and leading to tissue mixing and CSF contamination in the signal at white matter voxel locations. Additionally, the partial volume effects caused by the large voxels at this resolution reduces the mean FA. Blurring is similarly increased by the nonlinear registration needed to correct for motion and distortion. Furthermore, proximity to the noise floor where the Rician positive bias is significant compresses directional diffusivity differences by affecting the main eigenvalue disproportionately, making the diffusion tensor more isotropic (Jones and Basser, 2004).

Similarly, MD and RD values are overestimated. For example, literature suggests MD values of approximately 0.75 × 10^−3^s/mm^2^in the corpus callosum at 3 T (Hakulinen et al., 2012; Sener, 2001) and around 0.84 × 10^−3^s/mm^2^ in white matter as a whole. In this study, MD values in all tracts were between 1.05 × 10^−3^s/mm^2^ and 1.2 × 10^−3^s/mm^2^. This over-estimation can also be explained by partial volume effects and a broad point spread function leading to CSF contamination. A more detailed comparison of diffusion imaging between this 64 mT scanner and a high-performance 3 T system is presented in a recent publication (Gholam et al., 2026b).

The long echo train used by the non-cartesian TSE sequence, combined with the inherent low SNR and involuntary motion is responsible for the blurring and the signal dropout that can be seen in Figure 1. Further image degradation is introduced by gradient imperfections, which become more pronounced when moving away from the isocenter. Additionally, smaller tracts in the periphery of the brain may be smoothed out or buried in noise, whereas larger central tracts are more resistant to these effects because of their higher SNR. Together, these effects explain the drop off in the peripheral white matter in Figure 1, which is partly recovered in the population atlases shown in Figure 3.

### 4.2 Repeatability and reliability

The low wsCV and strong measurement agreement from the Bland-Altman analysis show that the measurements are precise and repeatable at the individual level. A similar analysis (Koller et al., 2021) with an ultra-strong gradient 3 T scanner found lower coefficient of variations for comparable tracts and metrics (between 0.2% and 1.0% as opposed to 2.2% and 4.4% in this study). In that analysis, standard deviations in the mean FA, MD, and RD values were approximately an order of magnitude lower than in this dataset.

A study of diffusion tensor imaging with a 0.55 T mid-field system with high performance gradients (Kung et al., 2024) also showed strong repeatability across white matter voxels, a more strict test than over a tract average. They obtained wsCV values of under 6% in FA and under 7% in MD. These results reflect the fact that mid-field is usually not a comparable regime to low-field. This mid-field system offers roughly 25 times increase in SNR due to *B*_0_ alone when considering the SNR $\propto B_{0}^{3/2}$ scaling suggested in low field MR physics review paper (Marques et al., 2019). This scaling, along with the higher performance hardware such as shielded high performance gradients and superconducting magnets offering excellent homogeneity compared to permanent magnet systems, provide access to higher resolution and more reliable imaging techniques.

ICC values were generally low due to the young population exhibiting minimal true inter-subject variability and CSF contamination pulling values toward a common mean, leading to between-subject variation becoming smaller than measurement variance, as shown by the negative lower bounds for the confidence intervals in Table 1. The wide confidence intervals further indicate the sample size was insufficient for stable ICC estimation. Intra-participant variance is susceptible to increases due to differences in tracking quality and overlap, since these tracts were extracted in the native space of each scan, and not validated by a neuro-anatomist. Consequently, part of the observed variance may reflect the stability of the tractography algorithm and the overall quality of the scans, rather than solely underlying biological variation.

### 4.3 Feasibility

Statistical power calculations show that effect sizes commonly reported in diffusion MRI studies are detectable with feasible sample sizes. For instance, in this study, the sample size estimates to detect a 4% difference in MD and RD were around 25 participants per group, and 65 participants per group to detect the same difference in FA. While feasible, power calculations at 3 T (Koller et al., 2021) show that detecting even a 1% difference in FA, MD, and RD in the CST would require under 10 participants per group, and up to 30 per group in the AF. The MD and RD sample size estimates in this dataset could also be optimistic because CSF contamination can reduce inter participant variability, artificially inflating statistical power.

Tractometry approaches are commonly used in addition to full tract averages in population studies since micro-structural changes may be situated in specific segments of tracts. This approach was not chosen here since performing and evaluating tractometry from low field diffusion data is an extra layer of processing and analysis that will be explored in further work.

Further sequence optimization and processing methods are required to shorten scan times and improve usability, particularly for clinical or developmental populations. Improved processing methods for shortening scan times by using fewer diffusion directions are being explored. These include but are not limited to: super-resolution methods (Mawuli Ametepe et al., 2025), deep learning synthesis approaches such as DeepDTI Tian et al. (2020), and joint reconstruction frameworks (Kung et al., 2024). These methods exploit correlations across diffusion volumes and have shown promise in recovering high angular resolution diffusion information from substantially reduced sampling schemes. Additional improvements in protocol duration could come from removing the need for the structural *T*_2_ image by using a different method to calculate response functions or obtain a white matter mask.

### 4.4 Conclusion

Our findings show that despite known low-field limitations, portable 64 mT MRI can provide repeatable diffusion tensor metrics across major white-matter pathways. With continued improvements in acquisition efficiency and image quality, low-field systems could support large, diverse, and geographically distributed cohorts that modern neuroscience increasingly demands. Establishing reliable diffusion measurements at low-field is a key step toward democratizing advanced neuroimaging and expanding global participation in brain-science research. The growing co-deployment of portable low-field and high-field scanners creates an opportunity to accumulate paired datasets large enough to learn cross-field-strength signal representations, potentially using high-field priors to improve low-field image quality. However, low-field measurements carry hardware and sequence-specific artifacts that differ across scanner configurations. Such cross-field-strength models are therefore likely to generalize best within a shared scanner and protocol family rather than across heterogeneous low-field hardware, which is a particularly tractable scenario where, as in this study, the same portable platform is deployed across multiple sites, including in LMICs. Repeatability studies of the kind presented here will be an important foundation for evaluating and validating any such harmonization approach as the global low-field MRI footprint grows.

## Data Availability

Raw MRI data cannot be shared because of ethical and participant confidentiality restrictions. Derived data supporting the conclusions of this article may be available from the corresponding author upon reasonable request and subject to institutional approval.
